# Symptoms and prevalence of common mental disorders in a heterogenous outpatient sample: an investigation of clinical characteristics and latent subgroups

**DOI:** 10.1186/s12888-023-05314-6

**Published:** 2023-11-03

**Authors:** Martin Brattmyr, Martin Schevik Lindberg, Jakob Lundqvist, Stian Solem, Odin Hjemdal, Frederick Anyan, Audun Havnen

**Affiliations:** 1 Department of Psychology, Norwegian University of Science and Technology (NTNU), Trondheim, NO-7491 Norway; 2 Mental Health Care Services, Trondheim Municipality, Norway; 3 Division of Psychiatry, Nidaros Community Mental Health Centre, St. Olavs University Hospital, Trondheim, Norway

**Keywords:** Patient-reported outcome measures, Latent class analysis, Factor mixture models, Common mental disorders, Comorbidity

## Abstract

**Background:**

Patient-reported outcome measures (PROM) provide clinicians with information about patients’ perceptions of distress. When linked with treatment and diagnostic registers, new information on common mental health disorders (CMHD) and service use, may be obtained, which might be useful clinically and for policy decision-making. This study reports the prevalence of CMHD and their association with PROM severity. Further, subgroups of self-reported symptoms of depression and anxiety were examined, and their association with clinician-assessed mental disorders, functional impairment, and service use.

**Methods:**

In a cohort study of 2473 (63% female) outpatients, CMHD was examined with pre-treatment scores of self-reported depression and anxiety, and the number of assessments and psychotherapy appointments one year after treatment start. Factor mixture modelling (FMM) of anxiety and depression was used to examine latent subgroups.

**Results:**

Overall, 22% of patients with a CMHD had an additional comorbid mood/anxiety disorder, making the prevalence lower than expected. This comorbid group reported higher symptoms of anxiety and depression compared to patients with non-comorbid disorders. FMM revealed three classes: “anxiety and somatic depression” (33%), “mixed depression and anxiety” (40%), and “cognitive depression” (27%). The anxiety and somatic depression class was associated with older age, being single and on sick leave, higher probability of depressive-, anxiety-, and comorbid disorders, having more appointments and higher functional impairment. Although the cognitive depression class had less somatic distress than the mixed depression and anxiety class, they reported more functional impairment and had higher service use.

**Conclusion:**

The results show that higher levels of somatic symptoms of depression could both indicate higher and lower levels of functional impairment and service use. A group of patients with high somatic depression and anxiety was identified, with severe impairment and high service needs. By gaining insights into CMHD factors’ relation with clinical covariates, self-reported risk factors of depression and anxiety could be identified for groups with different levels of aggravating life circumstances, with corresponding service needs. These could be important symptom targets in different groups of patients.

## Introduction

Patient-reported outcome measures (PROM) have been increasingly applied to encourage patient involvement [1]. PROM can be helpful in the diagnostic process of common mental health disorders (CMHD) [2] and is often implemented to facilitate service planning [3]. Since accessible quality indicators about patients’ needs often are rudimentary and lack diagnostic and symptomatic information [4], PROM data could help providers to facilitate person-centred services [1, 2].

Although linking PROM with register data is underutilized, important knowledge about patients may be obtained by connecting these sources of information [3]. One potential outcome of combining PROM- and register data is to increase the knowledge about specific groups of patients, such as those with comorbid disorders. Compared to patients with non-comorbid CMHD, patients with comorbidity have been associated with higher service utilization, higher symptom severity, and higher levels of functional impairment [5]. One PROM study that examined comorbidity in outpatient treatment showed that 45% of patients with an anxiety disorder had comorbid depression, 64% with post-traumatic stress disorder (PTSD) had a depressive disorder, and that comorbid depression was associated with higher symptom severity both before and after treatment [6]. Studies like these demonstrate the additive value of PROM data linked with diagnostic registers of CMHD, to provide a better understanding of specific diagnostic groups of patients.

PROM-data has the additional capability of providing organizations with information about patients useful for decision-making purposes and allocation of resources, however, aggregated data at this level is seldom published [7]. Thus, a potential of PROM data is the possibility offered to analyse symptom severity across the whole sample, capturing symptom heterogeneity beyond diagnoses. However, the great heterogeneity in mental health populations is complicated to address, and various modelling procedures exist—all with strengths and limitations. In a review by Feczko et al. [8], they commented upon clinical subtypes, dimensional, and computational models. Although clinical subtypes in diagnostic manuals could account for some heterogeneity, their descriptive approach to establishing nosology has been criticized. Dimensional models have been proposed as more suitable in psychiatric research; however, subtypes are often inevitable when multiple dimensional constructs are measured simultaneously. Therefore, with technical developments in recent decades, computational models have been increasingly applied [8].

Mixture model analysis has become a frequently utilized computational statistical framework to aggregate symptom heterogeneity into a smaller set of homogeneous groups [9]. One model specification, latent class analysis (LCA), has been used to analyse symptoms of depression and anxiety simultaneously, and class indicators have identified important sub-samples in non-clinical adult populations [10–17] and clinical adult populations [18, 19]. In the latter two studies, three class solutions were identified, labelled by a gradient of symptom severity, where the higher severity classes were more strongly associated with CMHD, and with a decreased association with mild and low severity classes.

LCA, however, has been criticized when applied to detect homogeneous subgroups of CMHD [20] because of the strict methodological requirements of local independence: class indicators should only depend on the latent classes and not correlate. This is an unrealistic requirement for symptoms of depression and anxiety due to their frequent co-occurrence. The advantage of factor mixture modelling (FMM) over LCA is that FMM does not assume conditional independence of latent classes [21] and therefore has been suggested to be more suitable in psychiatric research [9]. Applying FMM with class indicators of depression and anxiety has been conducted within general adult populations [22], and for people with a lifetime episode of depression [23], but rarely within a clinical mental health setting.

The scope of the current study was two-folded: first, to examine the one-year prevalence of CMHD in a diagnostic register and their association with patient-reported symptom severity in a large cross-sectional heterogeneous psychiatric outpatient sample. In accordance with previous research, we hypothesized comorbid mood and anxiety disorders to be frequently occurring and associated with higher symptom severity.

Since examining the sample based on clinician-assessed diagnoses could obscure other clinically relevant associations of symptom-homogenous clusters of patients, we continued to assess latent subgroups using FMM. Thus, the second aim was to identify homogeneous subgroups based on self-reported symptoms of depression and anxiety and analyse their association with clinician-assessed mental disorders, functional impairment, and service use. Due to the exploratory nature of FMM, the numbers of identified classes were not hypothesized a priori.

## Method

### Sample

Norwegian patients requiring non-urgent specialized mental health treatment are typically referred by their general practitioners to local psychiatric outpatient clinics for treatment. At one such clinic, electronic PROM data using the nine-item Patient Health Questionnaire (PHQ-9) [24], the seven-item Generalized Anxiety Disorder scale (GAD-7) [25] and the Work and Social Adjustment Scale (WSAS) [26] was collected for patients who started treatment between February 2020 and February 2022. All patients with a first assessment were invited to participate. Groups of patients with a primary diagnosis of obsessive-compulsive disorder (OCD), schizophrenia, substance abuse, and elderly patients, were treated at other specialized units. Out of 2519 patients who consented to participate, patients with missing information on all self-report questionnaires were excluded (*n* = 46). The final sample consisted of 2473 outpatients (79% of the invited participants; see Table 1). Out of the participating patients, 97% also had available register data with diagnostic and treatment information.

**Table 1:** Sample characteristics of outpatients (n = 2473), and comparisons between comorbid and non-comorbid disorders

	n (%)	PHQ–9 sum [95% CI]	GAD–7 sum [95% CI]
Female	1563 (63)	15.69 [15.41–15.98]	12.33 [12.10–12.57]
Male	910 (37)	14.80 [14.41–15.19]	11.14 [10.81–11.47]
Single	1277 (57)	15.88 [15.57–16.19]	11.82 [11.55–12.09]
Not single	977 (43)	14.80 [14.47–15.13]	11.97 [11.69–12.26]
Sick leave	719 (32)	16.41 [15.99–16.84]	12.82 [12.46–13.19]
Not sick leave	1520 (68)	14.97 [14.71–15.24]	11.55 [11.32–11.77]
**Diagnostic data**			
*Bipolar*	125 (5)	16.50 [15.51–17.50]	12.21 [11.38–13.03]
*Depression*	865 (36)	17.57 [17.24–17.90]	12.77 [12.46–13.08]
*PTSD*	245 (10)	16.04 [15.34–16.74]	12.30 [12.37–13.55]
*Anxiety*	613 (25)	15.67 [15.22–16.11]	13.31 [12.96–13.67]
*Somatization*	92 (4)	15.10 [13.96–16.24]	12.41 [11.50–13.31]
*Mixed anxiety depressive disorder*	55 (2)	14.09 [12.66–15.52]	11.47 [10.36–12.59]
*Comorbid*	333 (14)	18.14 [17.63–18.66]	13.85 [13.37–14.32]
*No CMHD or mixed anxiety depressive disorder*	832 (35)	13.78 [13.39–14.18]	10.62 [10.28–11.00]
**Comparisons between patients diagnosed with comorbid (** ***n*** ** = 333) vs. non-comorbid disorders**			
*Bipolar*	87 (70)	*t* = 2.16, *p =* 0.033	*t* = 2.17, *p =* 0.032
*Depression*	557 (64)	*t* = 2.31, *p =* 0.021	*t* = 5.53, *p* < 0.001^*^
*PTSD*	181 (74)	*t* = 4.45, *p* < 0.001^*^	*t* = 2.45, *p =* 0.026
*Anxiety*	359 (59)	*t* = 9.49, *p* < 0.001^*^	*t* = 3.30, *p =* 0.001^*^
*Somatization*	59 (64)	*t* = 3.60, *p =* 0.001^*^	*t* = 0.68, *p =* 0.499

*Note*: Frequency in valid percent. Comorbid disorder was defined as having a bipolar or depressive disorder and PTSD or anxiety disorder or somatization disorder. Diagnostic data were available for *n* = 2411 patients

^*^ Statistically significant with Bonferroni adjusted *p*-value at 0.01

### Measures

Demographic information was extracted for age, gender, self-reported relationship status, and work status before treatment started. Diagnostic information in accordance with the ICD-10 and the number of appointments was collected until one year after the self-reported symptom assessment (extraction date in March 2023). Diagnoses were manually clustered into bipolar, depressive, anxiety, PTSD, somatization, and comorbid mood/anxiety disorder. The comorbid mood/anxiety disorders were patients with bipolar/depressive disorder and PTSD/anxiety/somatization disorder. This is equivalent to the ICD-10 categorization of chapter F30 and F40-disorders. A diagnostic category was made for patients who were only diagnosed with mixed anxiety and depressive disorder (F412). If they had an additional CMHD, they were categorized into their respective CMHD.

PHQ-9 and GAD-7 were used to measure symptoms of depression and anxiety, respectively. Both instruments use a 4-point Likert scale ranging between 0 (not at all) and 3 (almost every day). Their psychometric properties have been widely tested, both internationally [27, 28] and in Norwegian outpatient populations [29, 30]. Although both instruments were created to screen for their respective disorders, they are commonly used to measure symptom severity and have been recommended for health outcomes measurement [31].

WSAS was used to examine the degree of functional impairment [26]. The scale has five items that are scored on a 9-point Likert scale from 0 (not at all) to 8 (very severely), with a maximum score of 40. Its psychometric properties are well established, including among Norwegian outpatients [32].

### Statistical analysis

Skewness ≥ 2, and kurtosis ≥ 4 were used as thresholds for examining normal distributions. Sum-scores of PHQ-9 and GAD-7 showed acceptable skewness and kurtosis, thus means (M) with standard deviations (SD) were used together with one-way analysis of variance (ANOVA) with Bonferroni post hoc test for comparisons between non-comorbid CMHD. Student t-tests were conducted for comparisons between non-comorbid and comorbid CMHD. For gender distribution between non-comorbid and comorbid disorders, Pearson’s *x*^2^ was used. Due to multiple comparisons, Bonferroni-adjusted *p*-values were applied.

Mplus version 8.8 was used as statistical software for mixture model analysis [33]. To make use of all available data, the full-information maximum likelihood was used with robust estimation (MLR). Missing items for the class indicators PHQ-9 and GAD-7 were below 0.1% and Little’s MCAR test indicated missing completely at random (*p* = 0.361). Owing to the overlap in assessing restlessness by one item each from the PHQ-9 and GAD-7, item 8 in the PHQ-9 was removed prior to the analyses. We retained a three-latent-dimensional structure, comprising a cognitive depressive-, a somatic depressive-, and an anxiety latent dimension, informed and validated in a previous study [29]. The analyses were performed in three stages following the recommendations by Clark et al. [21]. In the first stage, the latent dimensions of symptoms of depression and anxiety were verified through confirmatory factor analysis (CFA). Model fit was evaluated with the following indices: Standardized Root Mean Square Residual (SRMR) [34] and Root Mean Square Error of Approximation (RMSEA) [33] values less than 0.08 and values equal to or less than 0.06 (upper 90% CI close to or < 0.08) respectively, a Comparative Fit Index (CFI) and a non-normed fit index, Tucker-Lewis index (TLI) greater than 0.95 [35].

In the second stage, LCA was used to identify progressively higher numbers of latent classes to determine patient clusters of class membership, based on self-reported symptoms of depression and anxiety. The purpose of the LCA was to investigate the degree of heterogeneity in the sample and determine the highest number of classes for the FMM. The LCA was examined for one to five classes to determine the optimal model, using maximum likelihood estimation. To avoid local maxima solutions, 1000 random starting sets with 250 final stage optimizations were specified. For model fit indices, the Akaike information criterion (AIC) and Bayesian information criterion (BIC) were used together with the sample size adjusted BIC (aBIC), where lower values equal better fit. Entropy levels closer to 1 indicate greater classification accuracy. Significant levels of the Lo-Mendell-Rubin likelihood ratio test (LMR), adjusted LMR (aLMR), and Bootstrap likelihood ratio test (BLRT) were used as indicators for satisfying model fit. The optimal number of classes was determined according to the model fit indices and theoretical interpretability.

Finally, in the third stage, FMM was used to explore diagnostic class membership and the range of severity within and across diagnostic classes based on self-reported symptom data. Due to the exploratory nature of FMM, all model variations reported in the existing literature were estimated [21]. However, the third and fourth model variations recommended by Clark et al. [21] mostly produced inadmissible solutions as a result of estimating too many parameters compared to the first and second model variations, therefore only the first and second model variations are reported. In the first model variation, FMM-1, only the factor means were allowed to vary across classes while the factor loadings and item intercepts were constrained invariant across classes, indicating that symptoms of depression and anxiety are measured equally across classes. The factor covariance was fixed to zero to indicate no within-class heterogeneity for the symptoms. This model variation, FMM-1, is also called nonparametric factor analysis model [36]. Next, the factor variance in FMM-1 was freely estimated in each class for the estimation of FMM-2 and allowed for within-class heterogeneity in the levels of the symptoms. The factor means of the first class were fixed to zero for identification purposes, but freely estimated in the other classes. The FMM-2 is also called mixture factor analysis [36]. Once the best fitting model solution was identified, various covariates were included to (i) explain between-class heterogeneity by regressing class membership on age, gender, and relationship status, using the Mplus R3STEP option, and (ii) to determine how class membership predicts relevant outcomes using the DECAT option (i.e., work status, diagnosed with bipolar, depression, anxiety, PTSD, somatization, comorbid depression and anxiety, and mixed anxiety depression disorder), and the BCH option (i.e., number of assessment and psychotherapy appointments, and functional impairment). The effect sizes of differences between classes were calculated using Cramér’s V.

## Results

Overall, 63% (*n* = 1524) of the patients were diagnosed with a CMHD (bipolar, depressive, PTSD, anxiety, or somatization disorder). Further, 2% (*n* = 55) were registered with a mixed anxiety depressive disorder without a CMHD. Other frequent prevalent diagnoses for patients with no CMHD were attention deficit hyperactivity disorder (10%, *n* = 245) and personality disorders (4%, *n* = 106), and symptoms and signs involving emotional state (ICD-10 code R45, 21%, *n* = 496). The 1-year prevalence of comorbid CMHD differed somewhat between diagnostic groups. For patients diagnosed with bipolar disorder (*n* = 125), 25% also had a depressive disorder, 8% had PTSD, 17% had an anxiety disorder, and 6% had somatization disorder. For patients with a depressive disorder (*n* = 865), 4% were also diagnosed with bipolar disorder, 6% with PTSD, 28% with anxiety disorder, and 3% with somatization disorder. For PTSD (*n* = 245), 4% were diagnosed with bipolar disorder, 23% with depressive disorder, 9% with anxiety disorder, and 2% a somatization disorder. For anxiety disorders (*n* = 613), 3% had bipolar disorder, 39% had depressive disorder, 3% had PTSD, and 5% had somatization disorder. For patients with somatization disorder (*n* = 92), 8% had bipolar disorder, 30% had a depressive disorder, 7% had PTSD, and 32% had an anxiety disorder.

Overall, 22% of patients diagnosed with a CMHD had a comorbid mood and anxiety disorder. Correspondingly, 14% of all patients with diagnostic data had a comorbid mood and anxiety disorder. Patients with a comorbid disorder reported more anxiety than non-comorbid depression and anxiety disorder, and more depression than non-comorbid PTSD, anxiety, and somatization disorder (Table 1).

There were statistically significant differences in symptoms of depression between non-comorbid CMHD [*F*(4, 1019) = 20.17, *p* < 0.001]. Bonferroni post hoc test showed that non-comorbid depression had a higher mean (17.33, SD = 4.97) than PTSD (M = 15.19, SD = 5.90), anxiety (M = 14.05, SD = 5.64), and somatization disorder (M = 14.39, SD = 5.57). However, there were no significant differences in anxiety symptoms between non-comorbid CMHD [*F*(4, 1033) = 1.31, *p* = 0.265]. Gender distributions between comorbid and non-comorbid CMHD were not statistically significantly different when adjusted for multiple comparisons, with 33% being male.

### Factor structure results

One factor CFA of depression and anxiety showed unsatisfactory model fit (*x*^2^ = 3205.815, df = 90, *p* < 0.001; SRMR = 0.075; RMSEA = 0.118 [90% CI = 0.115, 0.122]; CFI = 0.762; TLI = 0.722), as was a two-factor model, consisting of a depressive and anxiety factor (PHQ-9 and GAD-7 respectively) (*x*^2^ = 1637.922, df = 89, *p* < 0.001; SRMR = 0.057; RMSEA = 0.084 [90% CI = 0.080, 0.087]; CFI = 0.882; TLI = 0.860). Separate analyses of the factor structures of GAD-7 and PHQ-9 revealed a two-factor – cognitive and somatic – structure of the PHQ-9. Thus, a 3-factor structure comprising (i) cognitive and (ii) somatic depression, and (iii) anxiety reached acceptable model fit (*x*^2^ = 872.436, df = 85, *p* < 0.001; SRMR = 0.046; RMSEA = 0.061 [90% CI = 0.058, 0.065]; CFI = 0.940; TLI = 0.926) with two error covariances.

### Latent class analysis results

Model fit indices for the LCA are presented in Table 2. The 1-Class model had the largest AIC, BIC, and aBIC, thus demonstrating the worst model fit. The LMR test, aLMR test, and BLRT in the 2-Class model solution all had *p*-values < 0.01, indicating to reject the 1-Class model solution in favour of a 2-Class model solution. Statistically significant *p*-values for the LMR and BLRT indicated that the current (*k*-class) model fitted the data better than the model with one less class (*k*-1 class). Results from comparing the 3-Class to the 2-Class model solution favoured a 3-Class model solution, which had lower criterion indices than the 2-Class model solution. Similarly, the 4-Class model and 5-Class model all had smaller criterion indices. Although the criterion fit indices showed that there was an improvement in model fit when comparing the 3-Class model solution to the 4-, and 5-Class model solutions, the deterioration in the entropy fit statistic for the 4-Class model solution was more pronounced, followed by the 5-Class model solution, which indicates that the 4- and 5-Class model solutions contain classes that are not clearly separated. Higher entropy values indicate that classes are easily distinguishable and distinctive, and as such favoured the 3-Class model solution which had a relatively high entropy value. The three classes were labelled as; high distress class (43%), moderate distress class (41%), and low distress class (16%) since they only differed by the degree of symptom severity.

**Table 2:** Latent class, latent class factor and factor mixture model of depression symptoms (PHQ-9) and anxiety symptoms (GAD-7)

Model	LL	*k*	Entropy	AIC	BIC	aBIC	LMR	aLMR	BLRT
**LCA**									
One-class	-51091.173	30		102242.347	102416.743	102321.425			
Two-class	-46643.901	46	0.890	93379.801	93647.208	93501.055	< 0.001	< 0.001	< 0.001
**Three-class**	**-45609.141**	**62**	**0.867**	**91342.283**	**91702.701**	**91505.711**	**< 0.001**	**< 0.001**	**< 0.001**
Four-class	-44927.061	78	0.852	90010.123	90463.552	90215.726	< 0.001	< 0.001	< 0.001
Five-class	-44466.280	94	0.856	89120.559	89666.999	89368.338	< 0.001	< 0.001	< 0.001
**FMM-1**									
Three-factor, two-class	-45977.979	63	0.879	92081.959	92448.190	92248.023	< 0.001	< 0.001	< 0.001
Three-factor, three-class	-45124.728	82	0.848	90413.456	90890.137	90629.603	0.116	0.116	< 0.001
Three-factor, four-class	-44513.440	101	0.844	89228.881	89816.013	89495.111	0.239	0.239	< 0.001
Three-factor, five-class	-44124.081	120	0.844	88488.162	89185.745	88804.475	0.239	0.239	< 0.001
**FMM-2**									
Three-factor, two-class	-43660.406	69	0.911	87458.812	87859.921	87640.692	0.240	0.240	< 0.001
**Three-factor, three-class**	**-43260.611**	**88**	**0.993**	**86697.222**	**87208.782**	**86929.185**	**0.194**	**0.194**	**< 0.001**
Three-factor, four-class	-42800.962	107	0.998	85815.924	86437.935	86097.970	0.165	0.165	< 0.001
Three-factor, five-class	-43114.548	126	0.787	86481.095	87213.557	86813.224	0.221	0.221	1.000

*Note*: AIC = Akaike information criterion. BIC = Bayesian Information Criterion. aBIC = Sample size adjusted BIC. LMR = Lo-Mendell-Rubin likelihood ratio test. BLTR = Bootstrap likelihood ratio test. In bold is the selected model

### Factor mixture model results

Results from the factor mixture model analysis are presented in the bottom part of Table 2. The FMM with the lowest criterion indices was the 3-factor, 4-Class FMM-2 model. However, one of the classes in this model solution turned out to be spuriously extracted from the data as it contained no respondents, so we examined the next lowest criterion indices —the 3-factor, 5-Class FMM-2 model. The LMR test, aLMR test, and BLRT in the 3-factor, 5-Class FMM-2 model solution all had *p*-values greater than 0.05, which indicates that this model solution should be rejected despite lower criterion indices. Therefore, we then considered a 3-factor, 3-Class FMM-2 model which had the second lowest criterion indices. Although the LMR and the aLMR tests did not show unequivocal support for this model solution, the BLRT had a *p*-value < 0.001, indicating that this model solution significantly fitted the data. The entropy value for this model solution was also high, indicating that there is a clear separation between distinguishable classes. Furthermore, the 3-factor, 3-Class FMM-2 model replicates the combined results from the CFA and LCA analyses.

Selecting the 3-factor, 3-Class FMM-2 model implies that the underlying symptoms are conceptualized equivalently and normally distributed within classes. In other words, the three classes are represented by normally distributed patterns of symptoms of depression and anxiety such that individuals within classes can have quantitatively different ranges of symptom severity. The criterion indices for the FMM-1 solution were much higher and therefore unsuitable for model selection. Additionally, the assumptions of the FMM-1 imply that all patients within a class are having the same levels of distress and that there is no within-class heterogeneity in symptoms of depression and anxiety. This is unlikely to be correct as symptom variation exists as well as the range of severity, consistent with our FMM-2 model solution. Since we assumed variations in symptoms of depression and anxiety as well as the range of severity, the 3-factor, 3-Class FMM-2 model was chosen.

### Interpretation of classes from the factor mixture model

**Fig. 1:**
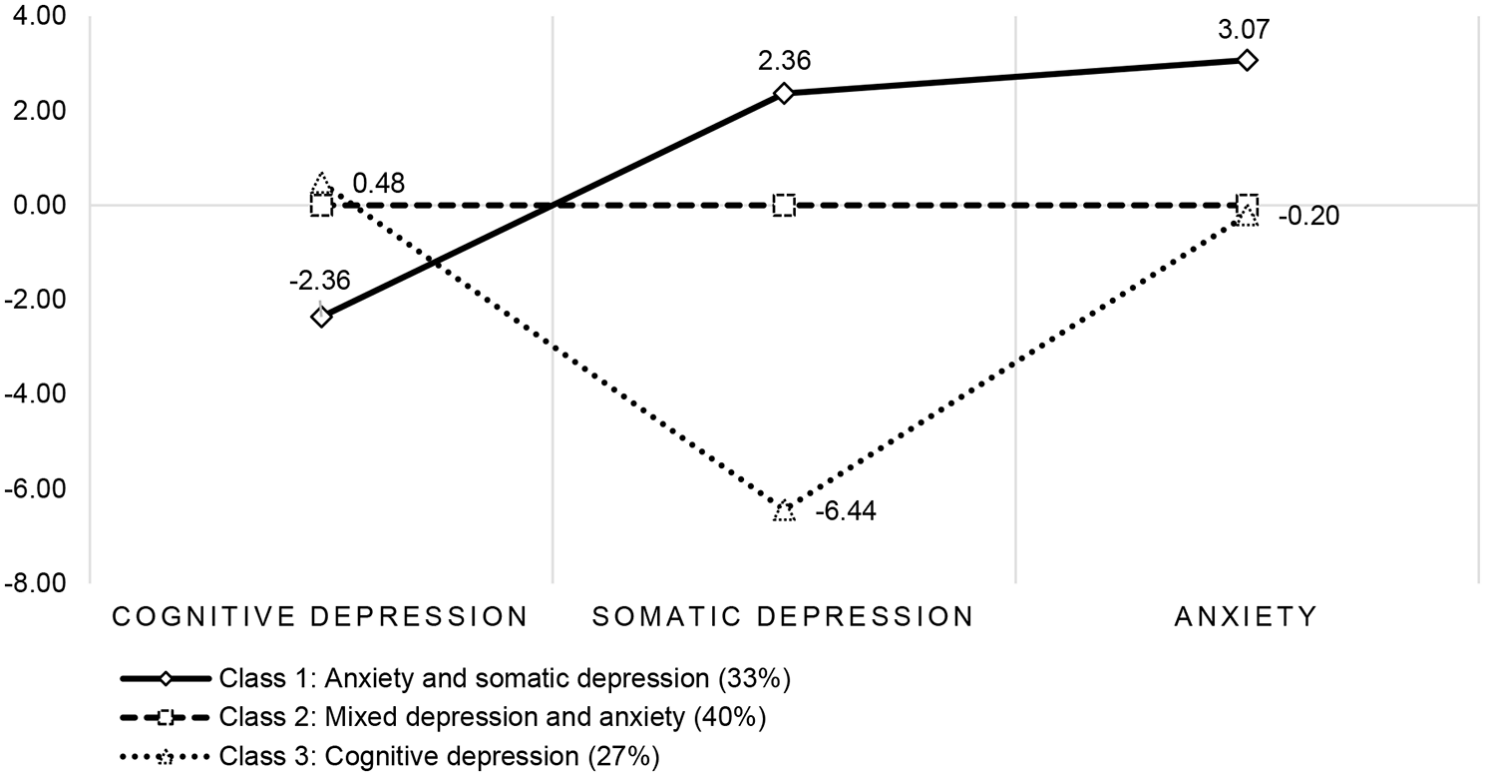
Three-factor three-classes factor mixture model latent variable factor means *Note*: Y-axis represents the factor mean in relation to the reference Mixed depression and anxiety class. The following dimension comprised class indicators; Cognitive depression: Loss of interest, sadness, worthlessness, and suicidal ideation. Somatic depression: Sleep problems, tiredness, appetite, and concentration. Anxiety: Nervous, not able to stop worrying, worrying too much, trouble relaxing, unable to sit still, annoyed and afraid

The three distress classes were labelled according to differences in factor means (see Fig. 1). The reference Class two comprising 40% of the sample, was labelled “*mixed depression and anxiety”* whereas patients in Class one (33%), in comparison to the mixed depression and anxiety class reported lower levels of cognitive depression, but higher levels of somatic depression and anxiety symptoms and was thus labelled *“anxiety and somatic depression”*. Patients in Class three (27%) on the other hand reported lower somatic depression than mixed depression and anxiety class and Class three was thus labelled *“cognitive depression”*. The factor variances within the classes were all significant, which agreed with the interpretation that there are variations in diagnostic class membership and the range of severity of the patient’s self-reported symptoms of depression and anxiety.

### Predictors of class membership

See Table 3 for predictors of class membership. There were no findings of gender predicting any class membership. Older age predicted a higher probability of membership to the anxiety and somatic depression class, compared to both the mixed depression and anxiety and the cognitive depression classes. Being single predicted a higher probability of membership in the anxiety and somatic depression class compared to the mixed depression and anxiety class, and the cognitive depression class compared to the mixed depression and anxiety class.

**Table 3:** Multinomial logistic regression parameters predicting class membership

Predictors	*OR*	*95% CI*	*OR*	*95% CI*
	Anxiety and somatic depression class		Cognitive depression class	
**Reference Class: Mixed depression and anxiety class**				
Females	0.92	[0.73–1.10]	1.05	[0.82–1.28]
Age	1.02^**^	[1.01–1.02]	1.00	[0.99–1.01]
Single	1.56^**^	[1.24–1.88]	1.27	[1.00–1.54]
	**Anxiety and somatic depression class**		**Mixed depression and anxiety class**	
**Reference Class: Cognitive depression class**				
Females	0.88	[0.68–1.07]	0.96	[0.75–1.17]
Age	1.01^**^	[1.00–1.02]	1.00	[0.99–1.01]
Single	1.23	[0.95–1.51]	0.79^*^	[0.62–0.96]

^*^p < 0.05, ^**^p < 0.01

### Differences in relevant outcome variables

Outcomes across classes are presented in Table 4. Patients with a high probability of membership in the anxiety and somatic depression class were to a larger degree associated with being on sick leave (*x*^*2*^ = 48.378, 6.569; V = 0.147, 0.054) and being diagnosed with depression (*x*^*2*^ = 146.772, 28.999; V = 0.256, 0.113), anxiety (*x*^*2*^ = 12.417, 1.651; V = 0.074, 0.027) and comorbid mood/anxiety disorder (*x*^*2*^ = 53.645, 7.788; V = 0.155, 0.059) compared to the mixed depression and anxiety class, and cognitive depression class respectively. Patients with a high probability of belonging to the cognitive depression class were to a larger degree associated with being on sick leave (*x*^*2*^ = 14.800; V = 0.081) and being diagnosed with depression (*x*^*2*^ = 29.255; V = 0.114), and comorbid mood/anxiety disorder (*x*^*2*^ = 15.442; V = 0.083), compared to the mixed depression and anxiety class. They also had a higher probability of being diagnosed with mixed anxiety and depressive disorder, compared to the anxiety and somatic depression class (*x*^*2*^ = 10.361, V = 0.068). Regarding the mixed depression and anxiety class, they had a higher probability of being diagnosed with somatization disorder (*x*^*2*^ = 5.149, V = 0.048) compared to the cognitive depression class. Regarding functional impairment and service use, the anxiety and somatic depression class had more assessments (*x*^*2*^ = 9.778, 0.652, V = 0.066, 0.017) and psychotherapy appointments (*x*^*2*^ = 133.021, 25.905; V = 0.244, 0.108) and reported the highest degree of impairment (*x*^*2*^ = 735.361, 206.953; V = 0.573, 0.304) compared to the mixed depression and anxiety class, and cognitive depression class respectively. The cognitive depression class reported more psychotherapy appointments (*x*^*2*^ = 35.389, V = 0.126) and functional impairment (*x*^*2*^ = 137.546, V = 0.248) than the mixed depression and anxiety class.

**Table 4:** Outcomes across classes

Outcomes (DECAT option)	1. Anxiety and somatic depression		2. Mixed depression and anxiety		3. Cognitive depression		Overall chi-square test	Sig. diff.	V
	*P*	95% CI	*P*	95% CI	*P*	95% CI			
Being on sick leave	0.414	[0.379–0.449]	0.252	[0.225–0.279]	0.313	[0.276–0.350]	48.383^***^	1 > 3 > 2	0.104
Bipolar disorder	0.047	[0.031–0.063]	0.050	[0.036–0.064]	0.060	[0.042–0.078]	1.145		0.017
Depressive disorder	0.546	[0.511–0.581]	0.193	[0.168–0.218]	0.375	[0.338–0.412]	270.687^***^	1 > 3 > 2	0.265
Anxiety disorder	0.289	[0.258–0.320]	0.239	[0.212–0.266]	0.235	[0.202–0.268]	7.133^*^	1 > 3,2	0.043
PTSD	0.103	[0.081–0.125]	0.106	[0.086–0.126]	0.093	[0.071–0.115]	0.732		0.014
Somatization	0.034	[0.022–0.046]	0.049	[0.035–0.063]	0.028	[0.016–0.040]	5.334	2 > 3	0.037
Comorbidity	0.228	[0.199–0.257]	0.075	[0.059–0.091]	0.121	[0.096–0.146]	79.783^***^	1 > 3 > 2	0.144
Mixed	0.011	[0.003–0.019]	0.022	[0.012–0.032]	0.038	[0.024–0.052]	11.173^**^	3 > 1	0.054
**Outcomes ** **(BCH option)**	**Mean**	**95% CI**	**Mean**	**95% CI**	**Mean**	**95% CI**			
Appointments									
*Assessment*	3.951	[3.675–4.227]	3.376	[3.147–3.605]	3.241	[3.008–3.474]	15.823^***^	1 > 3,2	0.057
*Psychotherapy*	10.854	[10.223–11.485]	6.267	[5.812–6.722]	8.215	[7.619–8.811]	134.160^***^	1 > 3 > 2	0.165
Functional impairment									
*WSAS score*	26.077	[25.609–26.545]	16.749	[16.245–17.253]	22.077	[21.556–22.598]	735.858^***^	1 > 3 > 2	0.386

*Note*: DECAT option is the probability of endorsing yes compared to endorsing no

Sig. diff = statistically significant differences between classes at *p* < 0.05 level in *x*^2^. CI = Confidence interval. V = Cramér’s V

^*^p < 0.05, ^**^p < 0.01, ^***^p < 0.001

## Discussion

This study shows that linking PROM with register data on CMHD can provide supplemental information on the service needs of routine mental health outpatients. By applying several procedures, different aspects of CMHD may be studied. In turn, clinicians can be made more aware of risk symptoms for difficult to treat patients, and policy decision makers informed of potential treatment targets. Patients with comorbid mood and anxiety disorders differed from non-comorbid patients as a group characterized by higher symptom severity. However, the prevalence of comorbidity (22%) was lower than what has been reported in previous studies [6], making further examination of the sample warranted. Thus, FMM-aggregated PROM data was examined. Three classes distinguished themselves regarding levels of cognitive depression, somatic depression, and anxiety, together with clinically relevant covariates. Since these classes had great within-class variability, patients within each sub-group were highly diverse. Nevertheless, by applying FMM, the simultaneous conceptualization of psychiatric constructs as dimensional and categorical could be made, which may provide a more parsimonious perspective of these complex constructs.

One potential of combining these sources of information is to examine the one-year prevalence of comorbid CMHD in psychiatric treatment. Since comorbid mood and anxiety disorders in Norwegian diagnostic registers have been suggested to be both overestimated [37] and underestimated [38], further examination was warranted. In a study by Torvik et al. [37], Norwegian national health register data was cross-validated with diagnostic interviews, showing a higher prevalence of comorbid mood and anxiety disorders in specialized mental health care registers compared to diagnostic interviews. On the other hand, Øiesvold et al. [38] examined the main diagnoses from medical case reports. They used cross-examination with an expert who had diagnostic interviews and hospital records available while being blinded to the diagnosis given in the case reports. Due to the lower prevalence of comorbid disorders in the case reports compared to expert opinions, Øiesvold et al. [38] concluded that comorbidity could be underdiagnosed in psychiatric registers. Consequently, comorbid mood and anxiety disorders could be under-communicated to the service suppliers, who risk to under-estimate the service needs of the patients. Therefore, we applied a third procedure, using all available registered mood and anxiety diagnoses one year after treatment started. In theory, this procedure could have inflated the prevalence of comorbid mood and anxiety disorder compared to point-prevalence. However, the low prevalence of comorbid mood-anxiety disorders compared to previous PROM research with diagnostic data [6] indicates that this was not an issue. The reason behind the low prevalence of comorbidity is unknown. Since data was extracted from a routine clinical setting, where clinicians have high caseloads, clinicians may be more concerned with reporting the primary diagnosis and less concerned with coding secondary diagnoses. This is in line with the assumption made by Øiesvold et al. [38] that clinicians rely on heuristic principles, which make them prone to not following up on seemingly irrelevant questions.

The second objective was to examine the characteristics of symptom-homogeneous subgroups. Similar to previous clinical LCA studies [18, 19], we also extracted three LCA classes, labelled high- moderate- and low distress classes based on self-reported depression and anxiety symptom severity. Due to the strict model assumptions of LCA, analysis with FMM was conducted. Since we assumed variations in symptoms of depression and anxiety as well as the range of severity, the 3-factor, 3-Class FMM-2 model was chosen over FMM-1, which additionally had worse criterion indices. The classes identified by FMM-2 reflected qualitatively different aspects of patients’ distress. The anxiety and somatic depression class had higher service use, higher levels of functional impairment, higher age, were more often single, and more often on sick leave, compared to the other classes. They had also higher rates of depression, anxiety, and comorbidity. It is therefore possible that this class, equal to one-third of the sample, was characterized by patients with persistent psychological problems of high complexity across a broad range of areas. The elevated levels of somatic-depressive problems together with anxiety reported by this class could therefore reflect a group of patients with a chronic course of depression. A longitudinal study on patients with depression showed higher cortisol levels and c-reactive proteins amongst patients with persistent problems, and these biomarkers had a considerably stronger relationship to somatic-, rather than cognitive symptoms of depression [39]. Thus, higher somatic symptoms of depression could indicate the chronicity of the disorder.

Levels of somatic symptoms of depression also separated the other two classes. The mixed depression and anxiety- were the largest class, compromising 40% of the patients. They were more often in a relationship and younger compared to patients in the other classes. Their levels of cognitive symptoms of depression and anxiety were similar to the class labelled cognitive depression class. Their higher levels of somatic symptoms of depression were unexpected since they also had less service use, reported a lower degree of functional impairment and lower levels of sickness absence. A possible explanation is that patients in the mixed depression and anxiety class are highly distressed but still able to work or study, which means that they are more exposed to daily stressors. These patients may express more somatic depressive complaints as a result of trying to cope with difficult everyday challenges. Correspondingly, patients in the cognitive depression class may be less exposed to work or study-related daily stressors, and at the same time report more severe functional impairment, and higher treatment needs.

The relationship between symptoms and functioning is complex, and many times weaker than expected. In a review, McKnight and Kashdan [40] argued that the dimensionality of the instruments and sample properties could obscure the relationship. Thus, when these are accounted for, new information may be obtained. Results showed the accumulated value of conjoining clinician-rated and self-reported information, as this provides new perspectives of the patient population that are not readily available from each source of information alone. Thus, a clinical implication of these findings is that higher symptom severity does not necessarily imply more service use and higher functional impairment when latent subgroups were accounted for. At the clinician-patient level, this finding shows the wariness one must have when interpreting patients’ symptoms of CMHD, since lack of somatic symptoms of depression could be associated with higher functional impairment. Further, by being informed of the severe impact high somatic symptom of depression together with anxiety could imply for difficult to treat patients, clinicians could become more aware of risk factors for resource demanding patients. The same information could also help policy decision makers evaluate treatments that target these factors for difficult to treat patients, and for patients at risk of deterioration. However, since the results are not unequivocal, alternative approaches for identifying subgroups might be better suited in clinical mental health contexts other than symptoms of CMHD, such as patients’ perceptions of functional impairment.

Our 3-factor 3-class FMM solution differed from two previous FMM studies examining depression and anxiety [22, 23]. Since the aims of the studies varied, differences could be derived from the population studied and assumptions of the underlying factor structure of applied class indicators. In a study examining the validity of subthreshold mixed anxiety and depressive disorder, a 2-factor 4-class solution was found, consisting of comorbid, depressive, anxiety and low symptom classes [22]. Another study, examining participants’ reports from their worst depressive episode in a general population subsample with a lifetime history of depression, specified a 1-factor 4-class solution, which was labelled severe depression with anxiety, moderate depression with anxiety, moderate depression without anxiety and mild depression [23]. Although objectives and results differed, these studies complement each other to show the additive value of examining depression and anxiety simultaneously.

Using FMM to aggregate PROM data, and to link such data with diagnostic registers could be used to combine two approaches to understanding psychopathology as dimensional (patients’ self-reported symptoms), or categorical (clinicians’ diagnostics) constructs. These perspectives may be viewed as complementary if analysed within an appropriate statistical framework. For example, Borsboom et al. [41] in a review of approaches to modelling the structure of psychiatric constructs, suggested that FMM may serve as a hybrid solution for modelling categorical and continuous approaches to measuring mental disorders at the same time. In line with this, we demonstrate that applying FMM to analyse PROM and register data may be justified in a context where dimensional phenomena such as symptoms of depression and anxiety are assumed to co-exist with categorical phenomena such as CMHD.

### Limitations

Some service indicators of interest were not available. The lack of longitudinal assessment is a limitation since this would allow for analysis of between-group trajectories and group transitions over time. This study did not account for dropouts, which could have informed service providers of groups at more risk of not completing their treatment. A broader spectra of patient information, such as previous treatment attempts, economic- and ethnic background could have added further information about the class results. Due to ethical constraints, we could not collect data on patients who declined participation, and there is thus a risk of selection bias. The diagnostic process was not conducted with research in mind and reflects the prevalence of CMHD given by clinicians with ordinary caseloads. Thus, the “true” prevalence could be higher than found in this study.

It is considered a strength that the sample was heterogeneous and included patients with broad spectra of distress that are usually found in routine outpatient treatment. However, since the sample contained not only patients with depressive or anxiety disorders, the use of instruments that measure symptoms of depression and anxiety could have missed other aspects of distress that patients might endure.

## Conclusion

PROM data is a valuable source of patient information, which has the potential to provide organizational knowledge on several levels when conjoined with patient data. However, due to the complexity of data, several procedures should be applied. This study used two procedures, first by making use of diagnostic data, showing prevalence of CMHD and associated clinical covariates, such as symptoms of depression and anxiety. Second, by aggregating PROM data with diagnostic and treatment registers using FMM, information about symptom-homogenous subgroups were obtained. This can be of use for understanding larger patient clusters, such as a group with high somatic symptom of depression and anxiety, with more severe functional impairment and higher service use. These patients were characterized by higher age and were more often single, indicating more aggravating life situations. This study also showed the caution that should be exercised when interpreting symptoms of CMHD, since their relationship with functional impairment and service use can be complex. Informed by class results and clinically relevant covariates, a group of patients had lower levels of somatic symptoms of depression, and at the same time higher levels of functional impairment, and higher service use than the least affected group.

However, since there is limited clinical research using FMM on anxiety and depression, and the research that’s been conducted shows ambiguous findings, the translational value of this procedure is still unclear. Additionally, since different factors of CMHD could be important targets in various groups of patients, further research is warranted.

## Data Availability

Data are available from the corresponding author upon reasonable request.

## Abbreviations

PROM: Patient-Reported Outcome Measures
CMHD: Common Mental Health Disorders
PTSD: Post-Traumatic Stress Disorder
LCA: Latent Class Analysis
FMM: Factor Mixture Modelling
PHQ-9: The nine item Patient Health Questionnaire
GAD-7: The seven item Generalized Anxiety Disorder scale
WSAS: The Work and Social Adjustment Scale
OCD: Obsessive-Compulsive Disorder
SD: Standard Deviation
ANOVA: Analysis Of Variance
MLR: Maximum Likelihood with Robust standard errors
CFA: Confirmatory Factor Analysis
SRMR: Standardized Root Mean Square Residual
RMSEA: Root Mean Square Error of Approximation
CFI: Comparative Fit Index
TLI: Tucker-Lewis Index
AIC: Akaike Information Criterion
BIC: Bayesian Information Criterion
aBIC: sample size adjusted Bayesian Information Criterion
LMR: Lo-Mendell-Rubin likelihood ratio test
aLMR: adjusted Lo-Mendell-Rubin likelihood ratio test
BLRT: Bootstrap Likelihood Ratio Test
